# Endogenous advanced glycation end products in pancreatic islets after short-term carbohydrate intervention in obese, diabetes-prone mice

**DOI:** 10.1038/s41387-019-0077-x

**Published:** 2019-03-11

**Authors:** Richard Kehm, Jana Rückriemen, Daniela Weber, Stefanie Deubel, Tilman Grune, Annika Höhn

**Affiliations:** 1 Department of Molecular Toxicology, German Institute of Human Nutrition Potsdam-Rehbruecke (DIfE), 14558 Nuthetal, Germany; 2 German Center for Diabetes Research (DZD), 85764 Muenchen-Neuherberg, Germany; 3 NutriAct-Competence Cluster Nutrition Research Berlin-Potsdam, 14458 Nuthetal, Germany; 4 German Center for Cardiovascular Research (DZHK), 10117 Berlin, Germany; 5 Institute of Nutritional Science, University of Potsdam, 14558 Nuthetal, Germany

## Abstract

Diet-induced hyperglycemia is described as one major contributor to the formation of advanced glycation end products (AGEs) under inflammatory conditions, crucial in type 2 diabetes progression. Previous studies have indicated high postprandial plasma AGE-levels in diabetic patients and after long-term carbohydrate feeding in animal models. Pancreatic islets play a key role in glucose metabolism; thus, their susceptibility to glycation reactions due to high amounts of dietary carbohydrates is of special interest. Therefore, diabetes-prone New Zealand Obese (NZO) mice received either a carbohydrate-free, high-fat diet (CFD) for 11 weeks or were additionally fed with a carbohydrate-rich diet (CRD) for 7 days. In the CRD group, hyperglycemia and hyperinsulinemia were induced accompanied by increasing plasma 3-nitrotyrosine (3-NT) levels, higher amounts of 3-NT and inducible nitric oxide synthase (iNOS) within pancreatic islets. Furthermore, *N*-ε-carboxymethyllysine (CML) was increased in the plasma of CRD-fed NZO mice and substantially higher amounts of arg-pyrimidine, pentosidine and the receptor for advanced glycation end products (RAGE) were observed in pancreatic islets. These findings indicate that a short-term intervention with carbohydrates is sufficient to form endogenous AGEs in plasma and pancreatic islets of NZO mice under hyperglycemic and inflammatory conditions.

## Introduction

Obesity and hyperglycemia, induced by nutritional overload and characterized by chronic inflammation, are among the major risk factors for the development of type 2 diabetes. Increasing evidence indicates that the formation of advanced glycation end products (AGEs), initiated and accelerated under hyperglycemic and inflammatory conditions, plays a crucial role in this metabolic axis^1,2^. AGEs and their precursors are compounds formed via nonenzymatic glycoxidation reactions, induced by the nucleophilic addition of free amino groups from proteins, lipids or nucleic acids to carbonyl groups of monosaccharides^1,3^. The so-called Maillard reaction leads to the generation of a reversible Schiff base adduct that spontaneously rearranges to Amadori products or reactive dicarbonyls^4,5^. During the advanced stage of the Maillard reaction, stable modifications such as *N*-ε-carboxymethyllysine (CML), *N*-ε-carboxyethyllysine (CEL) and amino acid cross-linking products such as pentosidine are formed^6^. AGEs-formation occurs exogenously during food processing or endogenously due to the presence of high amounts of reducing carbohydrates^7^. Exogenously generated AGEs are ingested with diets of which about 10% are absorbed by the body leading to an increase of circulating AGEs^8–10^. On the other hand, sustained elevated glucose levels trigger inflammatory processes, resulting in the endogenous generation of AGEs accelerated by high sugar consumption. High levels of AGEs were shown in plasma after glucose load in diabetic patients or after long-term intake of high carbohydrate diets in rat tissues, such as tail tendon and skin^11,12^. However, the short-term effects of carbohydrates on AGE-formation in pancreatic islets have not been yet investigated. Therefore, we determined the endogenous formation of AGEs in pancreatic tissue as well as plasma of diabetes-prone mice fed a carbohydrate diet for 7 days after carbohydrate restriction.

## Materials and methods

### Animal procedures and study design

Experiments were performed in male, obese and diabetes-prone New Zealand Obese (NZO) mice (NZO/HIBomDife mice, German Institute of Human Nutrition, Potsdam-Rehbruecke, Germany), housed in groups of four animals under standardized conditions (20 ± 2 °C, 12/12 h light/dark cycle) and had free access to food and water. Seven-week-old animals were randomly assigned into two groups (*n* = 8 per group). Group one received a carbohydrate-free, high-fat diet (CFD, 32% (wt/wt) protein and 31% (wt/wt) fat, C 1057-89, Altromin, Lage, Germany) for 11 weeks, the second group was additionally fed with a carbohydrate-rich diet for 7 days (CRD, 20% (wt/wt) protein, 28% (wt/wt) fat and 40% (wt/wt) carbohydrates), containing sucrose and starch, after carbohydrate restriction (for detailed diet compositions, see ref. ^13^). At the end of the trial, animals were sacrificed by acute exposure to isoflurane and blood and tissue samples were collected. Mice were kept in agreement with the National Institutes of Health guidelines for the care and use of laboratory animals. All procedures were verified and approved by the ethics committee for animal welfare of the State Office Environment, Health, and Consumer Protection (Germany, Brandenburg, 2347–21–2015). No sample size estimation was carried out.

### Body composition, blood glucose, and plasma analysis

Fat and lean mass of NZO mice were determined by nuclear magnetic resonance (NMR, EchoMRI™-100H, EchoMRI LCC, Houston, USA). Blood glucose levels were measured by using a CONTOUR® XT glucometer (Bayer, Leverkusen, Germany) immediately before sacrificing the mice. The concentration of plasma insulin and proinsulin was determined by performing a Mouse Ultrasensitive Insulin and Proinsulin ELISA (ALPCO, Salem, USA) according to the supplier’s instructions. Plasma 3-nitrotyrosine (3-NT) levels were determined with an in-house-designed ELISA (for detailed information, see ref. ^14^).

### Immunohistochemical analysis

Immunohistochemical labeling and quantitative analysis  was performed as previously described^15^. A list of used antibodies and applied concentrations are provided as supplementary information (SI Table 1). Imaging and quantification of all stainings were performed blinded; only the study leader had access to the code list.

### UPLC-MS/MS measurement

Sample preparation for protein-bound CML and CEL analysis in murine plasma as well as the detection via UPLC-MS/MS was performed as described in the SI. For determination of AGEs in the experimental diets, 25 mg of the diets were used and AGE content was measured following the procedure of CML and CEL plasma analysis.

### Statistical analysis

Statistical analysis was performed by using Graph Pad Prism version 7.04 (San Diego, USA). Group variances were similar and appropriate tests were performed to evaluate statistical differences. Shapiro−Wilk normality test was used to assess normal distribution. Accordingly, group comparisons were performed by two-tailed unpaired Mann−Whitney *U* test. All data are presented as mean values ± SD. Statistically significant differences were considered if *p* < 0.05.

## Results

**Fig. 1:**
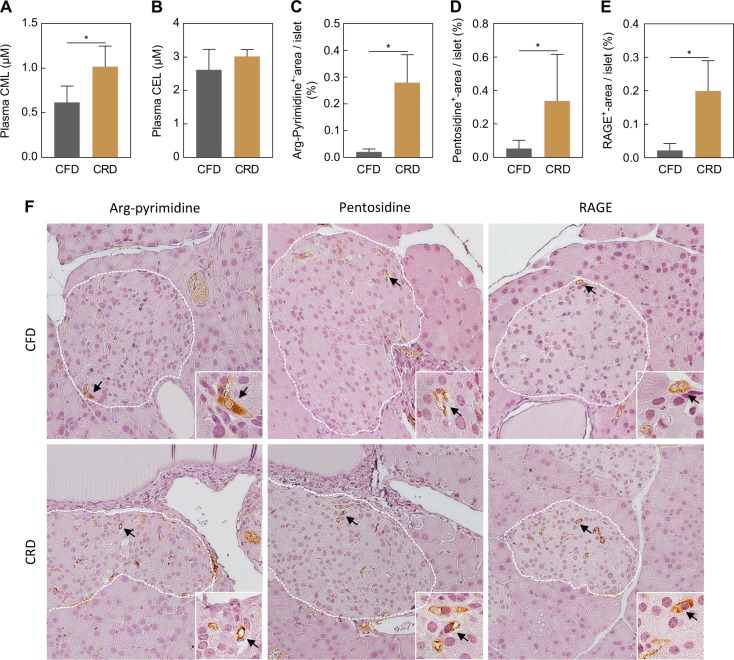
Short-term intervention with carbohydrates (7 days) induces the formation of AGEs in plasma and pancreatic islets of NZO mice. Plasma levels of CML (**a**) and CEL (**b**) in CFD- or CRD-fed animals measured by UPLC-MS/MS (*n* = 6). Quantitative analyses and representative images of immunohistochemical stained arg-pyrimidine (*n* = 8) (**c**, **f**), pentosidine (*n* = 8) (**d**, **f**) and RAGE (*n* = 8) (**e**, **f**). Images are represented in a magnification of 20x or 63x (inserts). White lines mark the islet area and black arrows indicate the positive-stained (brown) area. All results are presented as mean ± SD and statistical significance was assessed by Mann−Whitney *U* test (unpaired), *p < 0.05. AGE advanced glycation end product, CML *N*-ε-carboxymethyllysine, CEL *N*-ε-carboxyethyllysine, CFD carbohydrate-free, high-fat diet, CRD carbohydrate-rich diet, RAGE receptor for advanced glycation end products

Feeding a CFD for 11 weeks induced body weight gain and obesity in both experimental groups (Table 1—Body composition). Subsequent carbohydrate-challenge for 7 days increased blood glucose, insulin and proinsulin levels, indicating conditions of hyperglycemia and hyperinsulinemia. Moreover, plasma levels of 3-NT were almost threefold higher in the CRD group compared to the CFD group (Table 1—Plasma parameters). Quantitative analyses of immunofluorescent stainings revealed an increase of 3-NT and the inducible nitric oxide synthase (iNOS) by threefold and twofold, respectively, within the insulin-positive area of pancreatic islets in CRD-fed NZO mice (Table 1—Islet parameters, images not shown). Plasma analysis of protein-bound CML and CEL showed that CRD-challenged animals had 40% higher levels of plasma CML compared to the CFD group. In contrast, CEL plasma levels were unchanged but more than twofold higher in both groups compared to CML levels (Fig. 1a, b). Determining the AGE content of the experimental diets revealed higher CML-amounts in the CFD (CML: CFD 12.8 ± 1.9 mg/kg, CRD 6.2 ± 1.4 mg/kg), whereas the amount of CEL was higher in the CRD (CEL: CFD 28.4 ± 1.4 mg/kg, CRD 79.4 ± 1.2 mg/kg). As illustrated in Fig. 1c–f, immunohistochemical stainings revealed an eightfold increase of arg-pyrimidine in murine islets of CRD-fed NZO mice compared to the CFD group (Fig. 1c, f) accompanied by 5.2-fold higher amounts of the crosslinker-AGE pentosidine (Fig. 1d, f). Furthermore, the positive-stained islet area of the receptor for advanced glycation end products (RAGE) was ninefold higher in the NZOs challenged with carbohydrates (Fig. 1e, f).

**Table 1:** Body composition, plasma parameters, and inflammatory markers

	CFD	CRD
Body composition (NMR)		
Body weight (g)	79.93 ± 7.10	81.00 ± 4.99
Fat mass (g)	36.76 ± 3.06	37.19 ± 1.74
Lean mass (g)	34.36 ± 1.07	34.13 ± 0.92
Plasma parameters		
Glucose (mM)	7.58 ± 1.51	17.19 ± 3.38*
Insulin (nM)	0.72 ± 0.49	7.61 ± 3.67*
Proinsulin (nM)	0.07 ± 0.03	0.27 ± 0.09*
3-NT (pmol/mg)	3.36 ± 0.46	9.42 ± 3.32*
Islet parameters		
3-NT (%-area/ins^+^-area)	0.90 ± 0.59	2.80 ± 1.62*
iNOS (%-area/ins^+^-area)	0.54 ± 0.17	2.11 ± 0.99*

NZO mice received a CFD for 11 weeks and were fed subsequently with a CRD for 7 days (NMR *n* = 4, proinsulin *n* = 6, other experiments *n* = 8). All results are presented as mean ± SD and statistically significant differences were considered if *p *<* 0.05. CFD carbohydrate-free, high-fat diet, CRD carbohydrate-rich diet, iNOS inducible nitric oxide synthase, NMR nuclear magnetic resonance, 3-NT 3-nitrotyrosine

## Discussion

In the present study, we demonstrated that 7-day intake of a CRD, containing sucrose and starch, after preceding carbohydrate restriction is sufficient to increase the levels of protein-bound plasma CML as well as the amount of arg-pyrimidine, pentosidine and RAGE in pancreatic islets of hyperglycemic NZO mice. This reflects the high impact of short-term dietary sugars on AGE-formation in pancreatic islets.

It has been described that hyperglycemia in response to excess nutrients causes conditions of inflammation by the induction of multiple pathways linked to the formation of AGEs^3,5,7^. In accordance with this, we observed that NZO mice on a CRD develop hyperglycemia and hyperinsulinemia creating a proinflammatory environment, shown by an increase in plasma 3-NT levels and the amount of 3-NT and iNOS within the pancreatic islets. In addition, plasma levels of protein-bound CML, the reaction product of glyoxal and lysine, were increased after short-term feeding with carbohydrates. The lower amounts of CML in the CRD compared to the CFD indicate that higher plasma CML levels do not originate from the diet directly but were generated endogenously. In contrast, CEL-content of the CRD was higher but no differences were found in CEL plasma levels, indicating that neither carbohydrates nor dietary-derived AGEs had an influence on plasma CEL levels. In line with this, Schindhelm et al. found rising free plasma CML levels and unchanged CEL levels in type 2 diabetic women after a carbohydrate-rich meal^16^. By investigating the pancreatic islets of NZO mice an increase in methylglyoxal-derived arg-pyrimidine was observed after challenging with carbohydrates. Besides single-amino acid modifications, irreversible cross-linking of proteins represents another type of AGE-formation that is assumed to occur in at least weeks to months^17^. In contrast, we demonstrated that feeding a CRD for 7 days is sufficient to form cross-linked pentosidine, the product of the reaction between a pentose sugar with arginine and lysine residues in pancreatic islets of NZO mice. Furthermore, higher levels of AGEs and their precursors induce the expression of RAGE^1,18^. This is in accordance with our findings demonstrating that RAGE was increased within the pancreatic islets. The AGE-RAGE interaction initiates a signaling cascade leading to the activation of NFκB and NADPH-oxidase shown to trigger the generation of reactive oxygen species and oxidative damage^7,17^. Our study shows that although the measurement of plasma AGEs only reveals minor changes between CRD and CFD, murine pancreatic islets appear to be susceptible to glycation reactions. Increased glycation of pancreatic islets might promote the AGE-RAGE system and thereby lead to a vicious cycle of hyperglycemic damage. In conclusion, short-term intervention with a CRD for 7 days led to hyperglycemic and inflammatory conditions in obese NZO mice associated with the endogenous formation of AGEs in plasma and pancreatic islets.

## Supplementary information

Supplemental Material
