# Supplementary material for: Endogenous advanced glycation end products in pancreatic islets after short-term carbohydrate intervention in obese, diabetes-prone mice
Source: Nutr Diabetes. 2019 Mar 11;9:9. doi: 10.1038/s41387-019-0077-x (PMC6411991; doi:10.1038/s41387-019-0077-x)
Supplement: Supplementary file 1 — Supplemental Material [file 41387_2019_77_MOESM1_ESM.docx]

SI Table: Antibodies

| Antibody | Product # | Company | Dilution |
| --- | --- | --- | --- |
| Primary | | | |
| Rabbit polyclonal anti-nitrotyrosine | 06-284 | Merck Millipore | 0.5 µg/ml |
| Mouse monoclonal anti-3-nitrotyrosine [7A12AF6] | ab110282 | abcam | 1:200 |
| Rabbit monoclonal iNOS (D6B6S) | 13120S | Cell Signaling | 1:200 |
| Rabbit monoclonal anti-Insulin [EPR17359] | ab181547 | abcam | 1:10.000 |
| Mouse monoclonal insulin (L6B10) | 8138S | Cell Signaling | 1:200 |
| Mouse monoclonal methylglyoxal-AGE  (Arg-Pyrimidine) | AGE06B | BioLogo | 1:1000 |
| Mouse monoclonal pentosidine | PEN012 | BioLogo | 1:800 |
| Mouse monoclonal RAGE | sc-365154 | Santa Cruz | 1:100 |
| Secondary | | | |
| Goat anti-Rabbit IgG (H+L) Alexa Fluor 488 | A-11008 | Invitrogen | 1:200 |
| Goat anti-Mouse IgG (H+L) Alexa Fluor 633 | A-21052 | Invitrogen | 1:200 |

# UPLC-MS/MS measurements

## Sample preparation for CML and CEL analysis in plasma

Plasma protein-bound N-ε-carboxymethyllysine (CML) and N-ε-carboxyethyllysine (CEL) were analyzed by using UPLC MS/MS. 50 µl plasma were mixed with 100 µl of ultrapure water. Reduction of early glycation products such as fructoselysine was performed to avoid neo-formation of CML during heating in hydrochloric acid. Therefore, 250 µl sodium borate buffer (0.4 N, pH 10.2) and 250 µl sodium borohydride (1 M in 0.1 M NaOH) was added followed by incubation for 2h at room temperature. For protein precipitation, 1 ml of trichloroacetic acid (20%, w/v) was added followed by centrifugation (4 °C, 10000 rpm, 10 min). The supernatant was discarded, the precipitate was washed with trichloroacetic acid (5%, w/v) and the centrifugation step was repeated. After removal of the supernatant, 10 µl of internal standard (containing 20 µM ^2^H_4_-CML and 20 µM ^2^H_4_-CEL dissolved in water) and 1 ml of 6 M hydrochloric acid were added to the protein pellet and the sample was incubated at 110°C for 23 h. The hydrolyzed samples were evaporated to dryness with a vacuum concentrator (SpeedVac, Thermo Fisher Scientific, Schwerte, Germany). The residue was dissolved in 100 µl eluent B (10 mM ammonium formiate) and after centrifugation (4°C, 10000 rpm, 10 min) an aliquot of 90 µl was subjected to UPLC-MS/MS analysis. For a description of the UPLC MS/MS detection, see ESM methods.

## Ultra-High Performance Liquid Chromatography with MS/MS detection

UPLC analysis was performed with an Acquity Ultra Performance LC system coupled to a Waters Quattro Premier XE mass spectrometer (both Waters Corporation, Milford, USA). For chromatographic separation, an Intrada Amino Acid column at a column temperature of 40 °C was used. Solvent A was of 0.3% formic acid in ACN and solvent B was 10 mM ammonium formiate. The solvents were pumped at a flow rate of 0.6 ml/min in gradient mode (0 min, 0% B; 2 min, 0% B; 6.5 min, 40% B; 8 min, 90% B; 10 min, 100% B; 12 min, 100% B; 12.01 min, 0% B; 15 min, 0% B). The injection volume was 10 µl. Data were acquired and evaluated with the MassLynx Software (Waters, version 4.1). The ESI source was operated in positive mode and nitrogen was utilized as the nebulizing gas with a gas flow of 650 l/h and gas temperature of 350 °C. The capillary voltage was set to 2.5 kV, the source temperature was 150 °C and the cone voltage was 24 V. Analytes were measured in MRM mode with the following transitions and optimized collision energies (CE). Transitions used for quantification are labelled with q and transitions used for the confirmation of the presence of the analyte are labelled with Q. CML: 204.9🡪84.2 (q,CE: 18 V), 204.9🡪130.2 (Q,CE: 12 V); ^2^H_4_-CML: 209.2🡪88.1 (q, CE: 20 V), 209.2🡪134.1 (Q, CE: 12 V); CEL: 219.1🡪84.1 (q,CE: 18 V), 219.1🡪130.1 (Q,CE: 12 V); ^2^H_4_-CEL: 223.2🡪88.1 (q, CE: 20 V), 223.2🡪134.1 (Q, CE: 12 V). For quantification, external calibration with standard solutions in water containing CML, CEL and ^2^H_4_-CML, ^2^H_4_-CEL as internal standards was used. Calibration curves for CML and CEL, obtained by linear regression of a plot of the analyte/internal standard peak area ratio versus analyte concentration, were used to calculate concentrations in plasma samples. All plasma samples were analysed in duplicate.
